# Diabetes medication prescribing trends based on provider type and location in the United States

**DOI:** 10.1016/j.pmedr.2024.102947

**Published:** 2024-12-12

**Authors:** Susan D. Meeke, Megan M. Weemer

**Affiliations:** a A.T. Still University, 5850 E. Still Circle, Mesa, AZ 85206, USA; Novo Nordisk, Inc. 800 Scudders Mill Road, Plainsboro, NJ 08536, USA.; b A.T. Still University, 5850 E. Still Circle, Mesa, AZ 85206, USA

## Abstract

**Background:**

As primary care physician numbers continue to decline, more patients with type 2 diabetes are likely to receive care from advanced practice providers (APPs), including physician assistants and nurse practitioners. Analyzing diabetes medication prescribing trends among these provider types is essential for ensuring evidence-based diabetes care. **Purpose:** This retrospective, cross-sectional pilot study aimed to examine differences in type 2 diabetes medication prescribing trends by provider type (physicians vs. APPs) and geographic location, utilizing National Ambulatory Medical Care Survey (NAMCS) data.

**Methods:**

Data from the NAMCS were collected in August 2022 for the years 2015, 2016, 2018 and analyzed using IBM SPSS, employing chi-square analysis to assess associations between provider type, geographic location, and prescribed medications. Frequency distributions were calculated for patient characteristics and provider types.

**Results:**

Patients prescribed at least one diabetes medication (*N* = 1444) were included. Most received care from physicians (93.7 %) in metropolitan areas (82.8 %). Statistically significant associations were found between provider type, geographical location, and medications prescribed. Nurse practitioners were more likely to prescribe newer diabetes medications, while physician assistants frequently prescribed basal insulin. Patients in non-metropolitan statistical areas were more often prescribed older medications, whereas those in the West were less likely to receive older medications.

**Conclusions:**

The study revealed distinct prescribing patterns by provider type and geographic location. Notably, APPs tended to prescribe newer or specific medications in certain locations, highlighting the influence of provider type and geography on diabetes care. Further studies should include larger samples of APPs to deepen insights into these trends.

## Highlights

• Associations exist between provider type and diabetes medication prescribed.
• Nurse practitioners were more likely to prescribe a newer diabetes medication.
• Physician assistants were more likely to prescribe basal insulin.
• Older diabetes medications were prescribed more in small urban or rural areas.

## 1 Introduction

Diabetes is the seventh leading cause of death and a significant cause of disability and healthcare costs(Centers for Disease Control and Prevention, n.d.). Among patients with type 2 diabetes- the most prevalent form- various non-insulin medications are available, each class offering unique benefits for blood glucose control, weight management, and cardiovascular health. While metformin is typically the first-line medication, newer therapies provide advantages in hemoglobin A1c (A1c) reduction, weight loss, and cardiovascular benefits(American Diabetes Association, 2022). Despite their benefits, these newer treatments are underutilized in primary care, often due to cost, accessibility, or provider preferences 3,4.

Primary care physicians (PCPs) have historically managed diabetes care, serving as gatekeepers to evidence-based treatment. However, as the U.S. anticipates a shortfall of 49,300 PCPs by 2030, reliance on advanced practice providers (APPs)- nurse practitioners and physician assistants - will continue to grow, especially in rural and underserved areas(Smith et al., 2020; Frazier et al., 2022; Marcum et al., 2016; Morgan et al., 2017; Park et al., 2022). Studies have shown variations in prescribing patterns based on provider type and geographic region, which may affect patient outcomes. For example, Cipher et al.(Cipher et al., 2006) reported that while there were generally few prescribing differences among nurse practitioners, physician assistants, and physicians, rural nurse practitioners prescribed significantly more medications than their rural physician and physician assistant counterparts. Additionally, Marcum et al.(Marcum et al., 2016) and Park(Park et al., 2022) observed that APPs in rural areas prescribed more medications than physicians, suggesting a potential gap in access to newer treatments that could be crucial for optimal diabetes management.

Given APPs' increasing role in primary care, understanding specific differences in prescribing patterns between provider types is essential to ensure all patients benefit from evidence-based therapies, particularly those with newer agents that improve glycemic control and reduce complications(American Diabetes Association, 2022; Harris et al., 2021). Most existing studies aggregate APPs together(Marcum et al., 2016; Park et al., 2022; Federman et al., 2008; Kokoska et al., 2016), overlooking distinctions between nurse practitioners and physician assistants that may be clinically relevant. This study contributes to the literature by examining type 2 diabetes medication prescribing trends among physicians, nurse practitioners, and physician assistants in different geographic locations across the U.S., highlighting potential areas for improved training and resources to enhance diabetes care for all patients.

## 2 Methods

### 2.1 Sampling

The study used publicly available Centers for Disease Control (CDC) National Ambulatory Medical Care Survey (NAMCS) data from 2015, 2016, and 2018. Included were patients seen by physicians, nurse practitioners, or physician assistants in primary care with at least one diabetes medication. Excluded were those with type 1 diabetes, under 18, pregnant, or seeing specialists. The study was approved by the A. T. Still University Institutional Review Board.

### 2.2 Data collection

Of 51,450 participants in the NAMCS data sets, 1444 met the inclusion criteria after filtering in IBM SPSS Statistics.

### 2.3 Data analysis

Data analysis included frequencies and percentages for demographics, geographic location, and medication classes, as well as mean and standard deviation for medication count. Chi-square tests were used to assess associations between provider type, Metropolitan Statistical Area (MSA) status, geographic region, and medication categories/classes. Statistical significance was set at *p* < .05, and significant associations were further verified with post-hoc z-tests and Bonferroni adjustments. Metformin was excluded from the analysis, as it is a standard first-line therapy, while all other diabetes medications- including older oral agents, insulin, and modern non-insulin anti-diabetic drugs- were analyzed. The analysis included categories for physicians, physician assistants, and nurse practitioners, grouped under APPs, who are authorized to prescribe under a supervising physician's name (noted as a study limitation). All data were analyzed using IBM's Statistical Package for the Social Sciences (SPSS).

## 3 Results

### 3.1 Sample characteristics

The sample included 1444 patients on at least one diabetes medication from primary care providers: physicians (*n* = 1353; 93.7 %), nurse practitioners only (*n* = 3; 0.2 %), and physician assistants only (*n* = 2; 0.1 %) (Table 1). Some patients in the sample received a diabetes medication from both a physician and nurse practitioner or physician assistant. These groups are categorized as nurse practitioner/physician (*n* = 31; 2.4 %), or physician assistant/physician (*n* = 55; 3.9 %). Since nurse practitioners and physician assistants often prescribe under a physician, prescriptions in the nurse practitioner/physician group and physician assistant/physician group could have been written by the APP.

**Table 1:** Demographic characteristics and payor status of adult patients on at least one diabetes medication by provider type from the 2015, 2016, and 2018 national ambulatory medical care survey databases.

Characteristic	Physician	Physician assistant +/− physician	Nurse practitioner +/− physician	All providers
	N(/%)	N(%)	N(%)	N(%)
Gender				
Male	667(49.3)	28(49.1)	15(44.1)	710(49.2)
Female	686(50.7)	29(50.9)	19(55.9)	734(50.8)

Race/ethnicity				
Non-Hispanic White	962(71.1)	29(50.9)	27(79.4)	1018(70.5)
Non-Hispanic Black	160(11.8)	17(29.8)	0(0)	177(12.3)
Hispanic	146(10.8)	9(15.8)	7(20.6)	162(11.2)
Non-Hispanic Other	85(6.3)	2(3.5)	0(0)	87(6.0)

Type of payment				
Private insurance	512(37.8)	23(40.4)	16(47.1)	551(38.2)
Medicare	597(44.1)	18(31.6)	17(50.0)	632(43.8)
Medicaid/state based	120(8.9)	9(15.8)	1(2.9)	130(9.0)
Self-pay	25(1.8)	5(8.8)	0(0)	30(2.1)
No charge/charity/other	13(1.0)	0(0)	0(0)	13(1.0)
Blank/unknown	86(6.4)	2(3.5)	0(0)	88(6.1)

*Note*. Physicians were included in the totals with physicians and nurse practitioners due to the limited number patients (*n* = 5) who saw a physician or nurse practitioner exclusively.

Overall, there were 710 males (49.2 %) and 734 females (50.8 %). Most patients were non-Hispanic White (70.5 %). The nurse practitioner/physician group had higher percentages of female (55.9 %), non-Hispanic White (79.4 %), and Hispanic (20.6 %) patients. The physician assistant/physician group had more non-Hispanic Black patients (29.8 %;). Medicare was the most common payer (43.8 %), with the nurse practitioner/physician group having more private insurance (47.1 %) and Medicare (50 %).

Most patients lived in an MSA (82.8 %). Nurse practitioners/physicians were most likely to care for patients in non-MSA areas (44.1 %) and having more patients in the Northeast (47.1 %) and Midwest (23.5 %). Most patients were from the Northeast (25.6 %); few were from the South (10.8 %). Over a third lacked a designated region due to missing 2018 data (Table 2).

**Table 2:** Geographic characteristics of adult patients on at least one diabetes medication by provider type from the 2015, 2016, and 2018 national ambulatory medical care survey databases.

Characteristic	Physician	Physician assistant +/− physician	Nurse practitioner +/− physician	All providers
	N(%)	N(%)	N(%)	N(%)
Region of visits				
Northeast	336(24.8)	18(31.6)	16(47.1)	370(25.6)
Midwest	205(15.2)	11(19.3)	8(23.5)	224(15.5)
South	146(10.8)	8(14.0)	2(5.9)	156(10.8)
West	156(11.5)	8(14.0)	0(0)	164(11.4)
Unknown^a^	510(37.7)	12(21.1)	8(23.5)	530(36.7)

Metropolitan status				
MSA	1128(83.4)	48(84.2)	19(55.9)	1195(82.8)
Non-MSA	225(16.6)	9(15.8)	15(44.1)	249(17.2)

*Note.* MSA = metropolitan statistical area. Physicians were included in the totals with physician assistants and nurse practitioners due to the limited number patients (*n* = 5) who saw a physician assistant or nurse practitioner exclusively.

a Region data not collected by the National Ambulatory Medical Care Survey in 2018.

Patients averaged 1.6 diabetes medications (SD = 0.8), with the nurse practitioner/physician group averaging 1.9 (SD = 0.9). A total of 2262 diabetes medications were prescribed (Table 3).

**Table 3:** Frequencies of prescriptions for each diabetes medication class for all providers taken by adult patients with diabetes on at least one diabetes medication from the 2015, 2016, and 2018 national ambulatory medical care survey databases.

Medication class	Frequency
Metformin^a^	916
Older diabetes therapies	
Sulfonylureas^b^	345
Thiazolidinediones^b^	63
Glinides	10
Alpha-glucosidase Inhibitors	2
Modern non-insulin anti-diabetic drugs	
Dipeptidyl peptidase-4 inhibitors (DPP-4)^b^	191
Glucagon-like peptide-1 receptor agonists (GLP-1)	65
Sodium-glucose cotransporter-2 inhibitors (SGLT-2)^b^	81
DPP-4/SGLT-2 combination	3
Insulins	
Basal	369
Short-acting	202
Mixed insulins	14
Basal insulin/GLP-1 combination	1

*Note. N* = 2262. Percentages were not calculated due to patients being on more than one medication.

a Metformin is considered first-line therapy. ^b^Includes metformin combination pills.

### 3.2 Comparison of type 2 diabetes medications prescribed by provider type

Older medications (sulfonylureas, thiazolidinediones, glinides, alpha-glucosidase inhibitors) were prescribed 24.6 %–29.4 % of the time, with no significant association by provider type. However, a significant association was found for modern non-insulin anti-diabetic (MNIAD) drugs, with the nurse practitioner/physician group prescribing more in this category, χ2(2, *N* = 315) = 6.03, *p* < .05. No significant differences were found in insulin prescriptions by provider type, though physician assistants/physicians prescribed more insulin (42.1 %) than physicians alone (29.6 %).

When analyzed by medication class, no significant associations were found for sulfonylureas, thiazolidinediones (TZD), glinides, alpha-glucosidase inhibitors, dipeptidyl peptidase-4 inhibitors (DPP-4), sodium-glucose cotransporter-2 inhibitors (SGLT-2), combination DPP-4/SGLT-2, short-acting insulin, mixed insulins, or basal insulin/glucagon-like peptide-1 (GLP-1). All provider groups prescribed sulfonylureas 22.8 %–26.5 % of the time. Physicians prescribed TZDs more frequently (4.6 %). The nurse practitioner/physician group had higher rates for DPP-4 s (20.6 %), SGLT-2 s (11.8 %), and short-acting insulin (17.6 %). Nurse practitioners/physicians significantly prescribed GLP-1 s at a rate of 14.7 %, χ2(2, *N* = 65) = 9.3, *p* < .05. The physician assistant/physician group prescribed basal insulin more often (40.4 %) than physicians (24.9 %) and nurse practitioners/physicians (26.5 %), χ2(2, *N* = 369) = 6.87, p < .05.

### 3.3 Comparison of type 2 diabetes medications prescribed by geographic region

Patients in non-MSA areas were more likely to be prescribed insulin (33.3 %) and less likely to receive MNIAD medications (26 %), but these differences were not significant. A significant association was found between living in a non-MSA and receiving older diabetes therapies (33.3 % vs. 26 %, χ2(1, *N* = 394) = 5.55, *p* < .05).

Living in a non-MSA was significantly associated with being prescribed sulfonylureas (30.1 %, χ2(1, *N* = 345) = 6.42, p < .05). Non-MSA patients also had higher percentages for basal insulin (29.3 %) and short-acting insulin (16.5 %) compared to MSA patients. A slightly higher proportion of DPP-4 s were prescribed in MSA areas (13.6 %) compared to non-MSA areas (11.6 %).

Region data from NAMCS were only available for 2015 and 2016. A significant association was found between living in the West and not being prescribed older medications (18.3 %) compared to the Northeast (29.2 %), Midwest (28.6 %), or South (23.7 %), χ2(4, *N* = 394) = 9.57, *p* < .05. MNIAD medications were more commonly prescribed in the South (25 %) and West (25 %), but this was not significant. Insulins were more frequently prescribed in the West (34.8 %) and Midwest (31.3 %), but the difference was not significant.

By geographic region and medication class, living in the South (2.6 %) and West (2.4 %) was significantly associated with mixed insulin prescriptions, χ2(*N* = 13) = 11.3, p < .05, compared to the Northeast and Midwest. However, the small sample size may affect the validity of this finding. The West had lower sulfonylurea use (16.5 %) and higher basal insulin use (29.3 %). The Midwest had slightly more DPP-4 s (16.1 %) and short-acting insulin (14.7 %). The South had the highest SGLT-2 use (9 %). Overall, other medication uses were below 10 % for each class.

## 4 Discussion

Despite growing reliance on APPs in primary care, few patients in the NAMCS sample received care exclusively from nurse practitioners or physician assistants. Notable prescribing differences based on provider type and geographic variations were observed, although the 2018 data lacked specific geographic details. Most patients were non-Hispanic White, Medicare beneficiaries, and over 80 % were from urban MSAs. Among those with geographic data, the majority were from the Northeast.

Although APP numbers were low, a significant link was found between provider type and prescribing newer medications. The nurse practitioner/physician group prescribed more new medications, including GLP-1 s, than their physician and physician assistant/physician counterparts. This contrasts with findings by Marcum et al.(Marcum et al., 2016), who reported that physicians more likely to adopt new medications. The increased use of newer therapies by nurse practitioner/physician groups reflect a trend of refilling prescriptions initially written by physicians. Ellenbogen and Segal(Ellenbogen and Segal, 2020) suggest that nurse practitioners might be more influenced by pharmaceutical representatives due to their accessibility. Additionally, physician assistant/physician groups prescribed basal insulin, the second most common diabetes medication after metformin, reinforcing the notion that nurse practitioners and physician assistants are effective in managing chronic diseases(Morgan et al., 2017).

Geographic differences were assessed through MSA status and region. MSAs are urbanized areas with high social and economic integration(Bureau of Economic Analysis, 2023), while non-MSAs include smaller urban and rural populations(Centers for Disease Control and Prevention, 2023). Significant associations were found between living in non-MSAs and the prevalence of older diabetes therapies, possibly due to limited access to care. Sulfonylureas, an older medication class, were more frequently prescribed in non-MSAs, mirroring trends in rural Canada(Nagy et al., 2023). After metformin, sulfonylureas are the most commonly prescribed, and among the least expensive diabetes medications, making them more appealing for prescribers. Regionally, residents of the West exhibited lower usage of older therapies, with the highest prevalence in the Northeast and Midwest, possibly attributed to greater population densities(Raval and Vyas, 2020). The only significant association between medication class and region was mixed insulin use in the South, though this finding is questionable due to the small sample size (*n* = 13).

### 4.1 Study limitations and contributions

Despite a large sample size (*N* = 1444), the small number of nurse practitioners (*n* = 3) and physician assistants (*n* = 2) without a physician limited the analysis of solo prescribing. Visits involving multiple providers were categorized as nurse practitioner or physician assistant, which may not accurately reflect solo prescribing. NAMCS data may not fully capture prescribing differences by provider type(Jiao et al., 2018) and missing regional data for 2018 affected about a third of the sample, potentially impacting results for specific medication classes. Additionally, it was not possible to assess clinical inertia, patient health literacy or beliefs, medication costs, and physician peer influence in this analysis, as these are all factors impacting diabetes care.

As a pilot study, this research serves as an initial exploration of prescribing trends among three provider groups and supports existing research on geographic variations. It highlights the need for further investigation into individual prescribing practices and disparities. Future studies should explore databases reflecting individual prescriptions, compare new prescriptions by physicians with refills by APPs, and analyze geographic medication usage to uncover regional disparities and inform evidence-based care.

With a decline in practicing physicians, APPs are becoming increasingly crucial in primary care, especially in rural areas and in managing type 2 diabetes. While care quality appears consistent across physicians, nurse practitioners, and physician assistants(Smith et al., 2020; Jackson et al., 2018), this study confirms some noteworthy prescribing differences. Continued scrutiny of prescribing trends is essential for understanding current practices and informing policy changes that can enhance diabetes care quality across diverse patient populations. While access to care is a critical factor, we must also consider the roles of clinical inertia and provider education. How might these elements influence prescribing practices in different settings? What strategies can be implemented to enhance provider education in rural areas? Understanding these questions is vital for shaping future policies and educational initiatives aimed at optimizing diabetes management in diverse populations.

## Conflict of interest and funding source

The first author is currently employed as a Senior Diabetes Care Specialist by Novo Nordisk, Inc., with a business address of 800 Scudders Mill Road, Plainsboro, NJ 08536. The author wrote this article on her own behalf while a graduate student at A. T. Still University, and all information provided, and views expressed are those of the author and should not be attributed to Novo Nordisk, Inc. Novo Nordisk Inc. neither funded nor reviewed this article. There is no further duality of interest.

## CRediT authorship contribution statement

**Susan D. Meeke:** Writing – original draft, Methodology, Investigation, Data curation, Conceptualization. **Megan M. Weemer:** Writing – review & editing, Supervision, Methodology, Conceptualization.

## Declaration of competing interest

The authors declare the following financial interests/personal relationships which may be considered as potential competing interests: Susan Meeke reports a relationship with Novo Nordisk Inc. that includes: employment. If there are other authors, they declare that they have no known competing financial interests or personal relationships that could have appeared to influence the work reported in this paper.

## Data Availability

Data will be made available on request.
